# Machine Learning Models to Classify Shiitake Mushrooms (*Lentinula edodes*) According to Their Geographical Origin Labeling

**DOI:** 10.3390/foods13172656

**Published:** 2024-08-23

**Authors:** Raquel Rodríguez-Fernández, Ángela Fernández-Gómez, Juan C. Mejuto, Gonzalo Astray

**Affiliations:** Departamento de Química Física, Facultade de Ciencias, Universidade de Vigo, 32004 Ourense, Spain; raquel.rodriguez.fernandez@uvigo.gal (R.R.-F.); angela.fernandez.gomez@alumnos.uvigo.gal (Á.F.-G.); xmejuto@uvigo.gal (J.C.M.)

**Keywords:** origin labeling, sawdust block, stable isotope ratio, machine learning models

## Abstract

The shiitake mushroom has gained popularity in the last decade, ranking second in the world for mushrooms consumed, providing consumers with a wide variety of nutritional and healthy benefits. It is often not clear the origin of these mushrooms, so it becomes of great importance to the consumers. In this research, different machine learning algorithms were developed to determine the geographical origin of shiitake mushrooms (*Lentinula edodes*) consumed in Korea, based on experimental data reported in the literature (δ^13^C, δ^15^N, δ^18^O, δ^34^S, and origin). Regarding the origin of shiitake in three categories (Korean, Chinese, and mushrooms from Chinese inoculated sawdust blocks), the random forest model presents the highest accuracy value (0.940) and the highest kappa value (0.908) for the validation phase. To determine the origin of shiitake mushrooms in two categories (Korean and Chinese, including mushrooms from Chinese inoculated sawdust blocks in the latter ones), the support vector machine model is chosen as the best model due to the high accuracy (0.988) and kappa (0.975) values for the validation phase. Finally, to determine the origin in two categories (Korean and Chinese, but this time including the mushrooms from Chinese inoculated sawdust blocks in the Korean ones), the best model is the random forest due to its higher accuracy value (0.952) in the validation phase (kappa value of 0.869). The accuracy values in the testing phase for the best selected models are acceptable (between 0.839 and 0.964); therefore, the predictive capacity of the models could be acceptable for their use in real applications. This allows us to affirm that machine learning algorithms would be suitable modeling instruments to determine the geographical origin of shiitake.

## 1. Introduction

Shiitake (*Lentinula edodes*) is the second most common consumable mushroom in the world [1]. In addition, its powder can be used as a dietary supplement or a spice [2]. Over the past decade, cultivation has experienced exponential growth, taking second place in mushroom activity after *Agaricus bisporus* (J.E. Lange) Imbach [3], and represents around 22% of the total global mushroom production [4].

Shiitake mushroom is rich in nutrients and presents many minerals (K, Mg, and Mn, among others) and vitamins (pro-vitamin D_2_, B_1_, B_2_, and so on) [5]. Furthermore, it provides essential macro- and micronutrients, along with a wealth of bioactive compounds such as polysaccharides, antioxidants, and dietary fiber [5]. As a result, it offers consumers a range of nutritional and health benefits, from promoting health to disease prevention [5], among which can include anticaries potential and antigingivitic effect [6], immunomodulatory properties potential from their polysaccharides [7], anti-atherosclerotic bio-functionality potential [8], antimicrobial activity [9], thrombolytic and antithrombotic potential [10], among others. Nevertheless, according to Cheng et al. (2020) [11], fresh mushrooms are very perishable after harvesting due to different properties, such as their high respiration rate, among other characteristics [12]. Due to this, the distribution chain of this type of product is highly conditioned; hence, different technologies have appeared to ensure their sanitary aptitude [3].

In East Asian countries such as China, Japan, and Korea, shiitake mushrooms are usually consumed [4]. In fact, as far as global mushroom production is concerned, and according to Li et al. (2019) [13], China stands out as the largest producer (72% of the world’s production) of shiitake mushrooms, the most cultivated mushroom (25%) [14].

According to Chung et al. (2022) [15], in Korea, in particular, the sawdust blocks cultivation method is gaining popularity as an alternative to traditional log cultivation, as it offers several advantages, such as increased production performance and reduced cultivation time [16]. Several beginner shiitake mushroom farms commonly use inoculated substrates sourced from China. As a result, the cultivation of shiitake mushrooms using the sawdust-based method is a problem for a global standard for labeling due to this cultivation technique involves two different countries [15]. Consequently, it is frequently unclear whether these mushrooms have been grown using Korean or Chinese substrates [4]. With the growth of international food trade, the importance of knowing the origin of such products becomes relevant [17], especially to ensure food safety and prevent fraudulent practices related to origin labeling [17].

A commonly geodiscriminatory tool to track the origin of *Lentinula edodes* is stable isotope ratio analysis that can be used in combination with other techniques [17]. The use of machine learning methods, such as random forest (RF), support vector machine (SVM), and artificial neural network (ANN), represents a promising strategy for determining the geographical origin of shiitake mushrooms (*Lentinula edodes*) consumed in Korea.

According to Zhang et al. (2023) [18], the RF algorithm was proposed by Breiman [19] and can be used to solve classification and regression problems [20]. This kind of model is a highly flexible and powerful classifier based on decision tree models [21]. According to Chen et al. (2017) [22], an RF model generates distinct subsets of data from an original dataset using a bootstrap sampling method and subsequently constructs decision trees by training on these subsets. As a result, an RF is built by aggregating all these decision trees [22]. Integrating multiple classifiers reduces the variance and can produce more trustworthy results [20]. The result obtained in classification is determined based on the majority voting of these trees, while in the case of regression, an average of the results from each tree is obtained [22,23]. RF models are able to deal with incomplete data, correlated variables, or a huge number of input variables, among others [24]. RF finds applications in diverse research fields, such as in medicine to predict the occurrence of heart disease [25], in agriculture to examine the soil properties in semi-arid areas [26], in the atmosphere to forecast air quality [27], or in nanoscience to study the toxicity of titanium dioxide nanoparticles in biological networks [28], among others.

SVM is a machine learning method that, in agreement with Lipovina-Božović et al. (2019) [29], was introduced by Cortes and Vapnik (1995) [30]. In its elementary form, the support vector machine is a linear binary classifier that seeks to find an individual boundary between two classes [20]. According to Sheykhmousa et al. (2020) [20], linear SVM supposes that, in the input space, the multidimensional data are linearly separable. The SMV’s goal is to find an optimal hyperplane, using the training data, to split the dataset into a discrete number of classes [20]. On the other hand, it is also necessary to maximize the margin between the data train cases and the hyperplane to reduce the higher bound of the generalization error [31]. It should be noted that the points of the training data set near the separation hyperplane are named support vectors [32]. As it is well known, it is common for data samples from different classes to be not easily separable; for this reason, linear SVM classifiers cannot guarantee high accuracy when classifying this kind of data; thus, certain modifications are necessary [20]. This is where kernel functions come into play (linear function, radial basis function (RBF), among others) [33], which project the input dataset into a higher-dimensional feature space, allowing the training cases to be linearly separable [20]. SVM has the potential to be utilized in several research areas, including in food technology to detect and quantify bacteria pathogens in pork samples [34] or to identify edible oils according to their botanical origin [35], in nanomaterials to predict the adsorption capability of microplastics [36] or in pharmaceutical chemistry to drug design [37], and so on.

Last of all, ANNs are mathematical approaches inspired by the structure and functional characteristics of biological neural networks [38,39]. A neural network consists mainly of at least three layers [38,40]. In this sense, the network is fed through the input layer [41]. After this layer, there are the intermediate layers, called hidden layers, whose number depends on the complexity and the kind of calculations. Finally, there is an output layer where the ANN gets the outcome [41]. ANN exhibits a wide range of applications in numerous fields of research, like in food technology to develop different applications in winemaking technology [42], such as to control aging time in red wine [43], in biochemical engineering to study the transmembrane pressure in an anoxic–aerobic membrane bioreactor [44], in palynology to determine airborne Castanea pollen [45], in environmental sciences to determine monthly global solar irradiation [46], or inter alia.

In summary, in this research, three machine learning methods (RF, SVM, and ANN) were used to predict the geographical origin identification of shiitake (*Lentinula edodes*).

## 2. Materials and Methods

### 2.1. Experimental Data Used

The data used in this study were acquired through an experimental study conducted by Chung et al. (2021) [47]. In the study, fresh shiitake fruiting bodies were obtained by the authors from Korea’s mushroom farms or retail markets between 2017 and 2019. Only data for shiitake mushrooms produced using the sawdust block method have been considered for the development of machine learning models. According to this, the replicates were classified into three categories: Korean origin (125 replicates), Chinese origin (60 replicates), and Chinese inoculated sawdust blocks (93 replicates—from now on referred to as Chinese inoculated for abbreviation in writing). A Chinese inoculated case without δ^18^O value was deleted from the database.

Chung et al. (2021) [47] prepared the samples for stable isotope ratio analysis using a lyophilization process at −40 °C for three days and then pulverization to obtain a powder with particle sizes smaller than 400 µm. Then, the measurements of δ^13^C, δ^15^N, δ^18^O, and δ^34^S were determined.

### 2.2. Machine Learning Developed

Once the database was established, it was divided into three groups to carry out the machine learning process. The first group, the training set, which comprises 50% of the database, was used to train the different models developed. The validation group (30% of the database) was used to select the best model within all the models developed in the previous step. Finally, the testing group (20% of the database) was kept apart throughout the model development process to evaluate the final performance of the model and verify its ability to generalize its prediction power in external data.

These division percentages are based on the experience of previous models in which it has been shown that the training group always must be the majority, followed by the validation group and then, to a lesser extent, by the testing group. If the model achieves good results in this last group, the model could be considered capable of making accurate and reliable predictions in the phase of industrial use.

The first of the machine learning models developed was the random forest model. To do this, a bootstrap sample from the training dataset was used to build individual classification trees [48,49]. The set of decision trees combined form a forest that finally provides a result, in this case by confidence or majority.

In this research, different hyperparameters were used to identify the best RF model: (i) number of trees (from 1 to 100 with 99 steps), (ii) maximum depth (from 1 to 100 with 99 steps), (iii) the attribute selection criteria (gain ratio, information gain, Gini index and accuracy), (iv) pruning (setting up true or false), (v) pre-pruning (setting up true or false) and vi) voting strategy (confidence or majority vote). Models were developed with the values of their variables on a real scale, and models with normalized values have been developed using two methods: Z transformation (subscript Z) and range transformation among −1 and 1 (subscript R). This normalization was carried out with the training data, and later, this normalization model was applied to the validation and test data. The normalization process is carried out so that all the data of the variables used oscillate at a certain scale, avoiding, in theory, fluctuations that can cause unsatisfactory results.

The second model developed was the SVM model, which uses high-dimensional features [50] and is based on the theory of statistical learning [51]. The main advantage of this model lies in the kernel trick, which allows simultaneous minimization of both the model’s complexity and prediction error [52].

In this research, two types of SVM were used: C-SVM and nu-SVM. Additionally, three parameters were defined, where two of them were set according to the guide of Hsu et al. (2003) [53]: gamma (from approximately 3.05·10^−5^ to 8 with 18 steps), C (from 0.03125 to 32,768 with 20 steps), and nu (in the range of 0.1–0.4 with 3 steps). Additionally, data were normalized using Z transformation (subscript Z) and range transformation (−1 to and 1, subscript R). Moreover, gamma and C were performed on a linear and logarithmic (subscript L) scale.

An ANN consists of a structure of neurons, also called nodes, which are interconnected by links [54]. According to Hategan et al. (2021) [54], each unit receives information from its input links and then transmits the obtained result to its output links. Each of these links contains a numerical value that represents the weight between two neurons [54]. Backpropagation is widely used to train ANNs; basically, this algorithm seeks to minimize the error at each iteration by modifying the weights [55].

In this research, a multilayer perceptron (MLP) is composed of three layers (input layer, hidden layer, and output layer). The input nodes were δ^13^C, δ^15^N, δ^18^O, and δ^34^S. In the case of the hidden layer, the number of intermediate neurons was selected using the criterion 2*n +* 1, “*n*” being the number of input variables; therefore, the number of hidden neurons was in the range of 1 to 9. Finally, the output layer was one, and depending on the model being performed, the number of output variables was two (Korean origin and Chinese origin) or three (Korean origin, Chinese origin, and Chinese inoculated). The hyperparameters used for each ANN model were training cycles (from 1 to 524,288 with 19 steps on a linear and logarithmic scale, subscript L) and decay (setting up true or false). In addition, some models were normalized again using Z transformation (subscript Z) and range transformation (−1 to and 1, subscript R).

**Figure 1:**
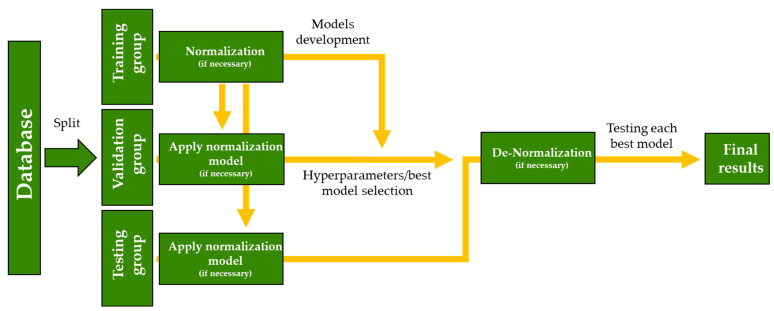
Flowchart for a general machine learning process.

A general flowchart for the development of the different machine learning algorithms used in this research is shown below (Figure 1).

### 2.3. Statistics Measurements

In this study, different statistical parameters were used to carry out the classification. On the one hand, accuracy and kappa statistics and Cohen’s kappa [56] (for binary classification) were used. The accuracy indicates the percentage of correct predictions, while the kappa and Cohen’s kappa also consider correct predictions that occur by chance. On the other hand, a binary classification was performed as the output variable could be of two types: Korean (positive class) and Chinese (negative class).

### 2.4. Software

A computer with an AMD Ryzen 7 1700X Eight-Core Processor with 32 GB of RAM and an operative system of Windows 10 Pro were used to develop the models. Data were collected using Microsoft Excel 2013 (15.0.5589.1000) from Microsoft Office Professional Plus 2013 and Excel version 2407 from Office 365. The different models were developed using the software RapidMiner Studio Educational, version 9.10.011 (RapidMiner GmBH, Dortmund, Germany). The different figures were made using PowerPoint version 2407 from Office 365 and SigmaPlot 13.0.

## 3. Results and Discussion

In this point, the results obtained by each of the three selected models for each study group will be analyzed in detail. Each model was selected among its peers using the highest value of accuracy in the validation phase. Subsequently, the operation performance of each model in a real environment will be verified using the external database.

### 3.1. Geographical Origin of Shiitake Mushrooms (Korean Origin, Chinese Origin, and Chinese Inoculated)

In this section, different models were developed to determine the origin of shiitake mushrooms in three categories (Korean, Chinese, and Chinese inoculated—125, 60, and 93 replicates). The first group of models that were carried out were the RF models. The three RF models (RF, RF_R,_ and RF_Z_) developed presented the same accuracy and kappa values in the training, validation, and testing phases. It seems clear that the normalization process in a random forest model is not very important, at least in this specific case. In this case, the RF model was selected since it was the first one developed. The selected RF model showed an accuracy of 0.940 and a kappa value of 0.908 for the validation phase (Table 1).

Furthermore, it can be observed from Table 1 that the selected RF model performs better during the training phase than in the validation phase, with an accuracy value of 0.971 and a kappa value of 0.954.

The second selected model developed is an SVM model. In this case, three linear SVM models (SVM, SVM_R,_ and SVM_Z_) and three other SVM models using a logarithmic scale (SVM_L_, SVM_R-L,_ and SVM_Z-L_) were developed. All these models achieved the same accuracy in the validation phase (0.928); nevertheless, a difference between them lies in the kappa value of the validation phase. In this case, the best model among all the SVM models developed was selected according to the highest accuracy value and the highest kappa value. Consequently, the best model was the SVM model, which showed a kappa value of 0.890. This good performance is also reflected in the training phase, showing better values of accuracy (0.993) and kappa (0.988).

Finally, the last group was the ANN group, when different ANN models were created using a linear scale (three models, ANN, ANN_R,_ and ANN_Z_), and three other models (ANN_L_, ANN_R-L,_ and ANN_Z-L_) were developed using a logarithmic scale. Normalized models on both scales showed accuracy values between 0.892 and 0.940, the ANN_R_ model being the one with the higher accuracy in the validation phase. In the training phase, the ANN_R_ model achieved an accuracy value of 0.971 and a kappa value of 0.954, providing a superior performance compared to the validation phase.

Once the three selected models have been presented, the test phase adjustments will be described and provide an idea about the predictive capacity of the model during its real use. The selected RF model obtained a lower accuracy value of 0.839 and a kappa value of 0.742. Although this accuracy value clearly decreases with respect to the training and validation phases, it can be said that an accuracy value of 83.9% could be acceptable. The SVM model, which presented for the validation phase, a close accuracy value (0.928) that the RF model (0.940), presents for the test phase a slight improvement accuracy (0.857, with kappa of 0.774) concerning the results offered by the RF model (0.839, with kappa 0.742). According to the adjustments, the best model in the testing phase is the ANN_R_. This assertion is supported by observing the adjustments presented in the testing phase (0.857 and 0.774, accuracy and kappa values, respectively, the same performance obtained by the SVM model) and the accuracy value in the validation phase (0.940, with a kappa of 0.908, a very slightly higher kappa value for the RF model).

Considering that the RF model presents the highest value of accuracy and the highest kappa value for the validation phase, this model is chosen as the best model of the three selected models, despite that in the testing phase, the model showed slightly lower results than the other two models.

**Figure 2:**
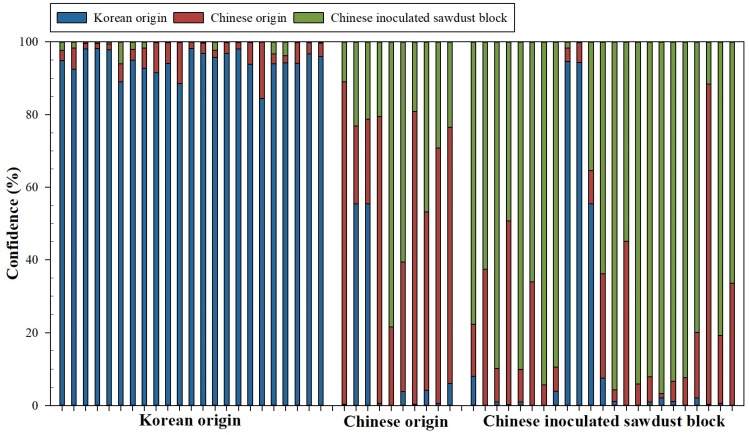
Confidence values (%) for test replicates obtained by the RF model.

Figure 2 shows the confidence level for the testing replicates using the RF model. The testing phase is made up of 56 replicates (23 Korean origin, 10 Chinese origin, and 23 Chinese inoculated).

For the 23 Korean origin replicates, the selected RF model has correctly predicted all the samples. Within the replicates, 10 were predicted with confidence values above 95.0%, and the other 13 replicates were predicted with confidence values between 89.0% and 94.9%. For the Chinese origin replicas, five of them have been correctly classified with confidence values above 70.3% and the other one with 49.1%. The four remaining replicas have been incorrectly classified as Chinese inoculated (two replicates—60.6 and 78.4% for the confidence for this class) and Korean origin (two replicates—both with 55.4% for the confidence for this class). Finally, for 23 Chinese inoculated replicates, 8 of them have been correctly classified with confidence values greater than 90.0%; the other 10 replicates showed confidence values between 54.9% and 89.9%. Within the last five replicates, two replicates were classified as having Chinese origin with confidence values, for this class, of 50.5 and 88.1%. The other three replicates were classified as Korean origin class with confidence values of 55.4, 94.4, and 94.6%.

The confidence matrix of the RF model for the test phase and all data used are shown below (Table 2). As can be seen, the RF model works satisfactorily for the test phase, presenting an accuracy of 100 and 78.3% for the Korean and Chinese inoculated categories, respectively. In the case of the Chinese origin category, the accuracy decreased to 60.0%. For all data, the RF model presented an accuracy of 98.4 and 94.6% for the Korean and Chinese inoculated categories, respectively. Again, the Chinese origin category was the one that presented the lowest accuracy (81.7%). The accuracy for all data sets was 93.5%, with a kappa value of 0.898.

These results could be compared with the data obtained from the article of Chung et al. (2021) [47] (Table 2). In that article, a source identification model established on stable isotope ratio analysis joined with discriminant analysis was used, obtaining an accuracy of 78.4% for the original set, while in this study, a higher accuracy value of 93.5% was obtained by the best model (RF). It can be concluded that the best model, based on the validation results and the rest of the selected models, could be suitable models to determine the geographical origin of shiitake mushrooms cultivated in sawdust block.

### 3.2. Geographical Origin of Shiitake Mushrooms (Korean Origin and Chinese Origin-2)

In this section, distinct models were developed to determine the origin of shiitake mushrooms in two categories: Korean origin (125 replicates) and Chinese origin-2 (153 replicates from Chinese origin and Chinese inoculated). In other words, in this case, the category of Chinese origin is made up of Chinese origin replicates and the Chinese inoculated replicates, and for model development, they will be considered as a single category.

As happened in the previous section, the three RF models (RF, RF_R_, and RF_Z_) have returned to show the same accuracy and kappa values in the training, validation, and testing phases. This could indicate, in the same way as in the previous section, that the normalization process is not an important stage in the development of RF models, at least in this research case. The RF model showed, during the validation phase, a 0.976 and 0.950 for accuracy and kappa values (Table 3). For the training phase, the RF model presents slightly better adjustments, increasing to an accuracy of 0.986 and a kappa value of 0.971.

The six SVM models developed present, for all configurations, accuracies of 0.988 and kappa values of 0.975. To select the best model among them, it was necessary to look at the training and validation phase adjustments, where the SVM exhibited the highest accuracy (0.991) with a kappa value of 0.982. Finally, the ANN models showed the same accuracy for the validation phase (0.976), except the ANN_L_ presented an accuracy value of 0.964. It was necessary to observe the kappa value to select the best neural model, which is, in this case, the ANN_R_, which showed a kappa value of 0.951. These high values are also reflected in the training phase. Among the three selected models presented in Table 3, it can be said that the best model is ANN_R_, which achieved an accuracy of 0.982 in the joined training and validation phases.

Once the three selected models have been analyzed, it is essential to consider the testing phase to check for good generalization performance. In this regard, the ANN_R_ models exhibit an accuracy value of 0.893 in the testing phase (Table 3). These accuracy values could be acceptable, taking into account the accompanying kappa value (0.787), which places in the upper part of the substantial level of the strength of agreement (according to the division established by Landis and Koch (1977) [57]. The other selected models, RF and SVM models, showed a high accuracy value (0.929 and 0.964, respectively). Given these results, it could be said that the SVM model, in general, for any of its phases, presents high accuracies (greater than 0.964) and high kappa values (greater than 0.927).

Comparing the three selected best models, the SVM model is chosen as the best model of the three selected models because it presents the highest value of accuracy (0.988) and the highest kappa value (0.975) for the validation phase.

**Figure 3:**
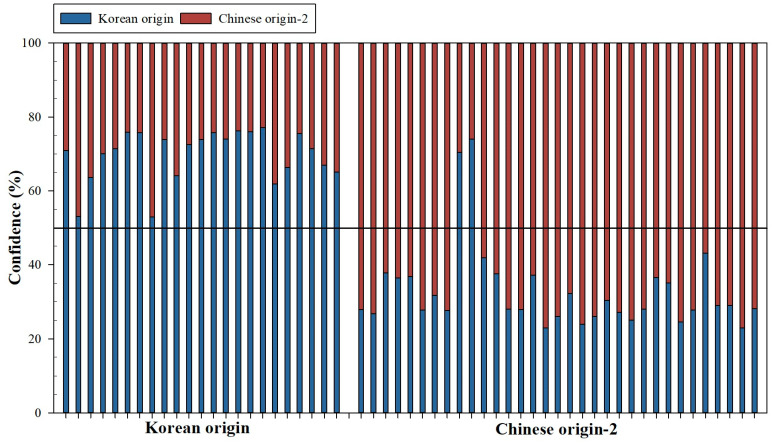
Confidence values (%) for test replicates obtained by the SVM model.

Figure 3 shows the confidence level for the testing replicates using the SVM model. The testing phase is made up of 56 replicates (23 Korean origin and 33 Chinese origin-2).

Of the 23 Korean origin replicates, all of them have been correctly classified, 7 of them with confidence values greater than 75.0% and the other replicates with confidence values between 53.0 and 74.0%. Concerning the 33 Chinese origin-2 replicates, it should be noted that 31 have been correctly classified (19 of them with confidence values greater than 70.0% and 12 of them with values between 56.9 and 70.0%). The rest of the replicates of Chinese origin (2) have been incorrectly classified with confidence for Korean origin values of 70.5 and 74.1%.

The confidence matrix of the SVM model for the test phase and for all data used are shown below (Table 4). As can be seen, the SVM model works well for the test phase, presenting an accuracy of 100.0 and 93.9 % for the Korean and Chinese origin-2 categories, respectively. For all data, the SVM model presented an accuracy of 99.2 and 98.0% for the Korean and Chinese origin-2 categories, respectively. The accuracy for all data sets was 98.6%, with a kappa value of 0.971.

These results could be compared with the data obtained from the article of Chung et al. (2021) [47] (Table 4), showing that the SVM model obtained higher accuracy (98.6%) than the original model (93.5%) proposed by Chung et al. (2021) [47] for the original set. According to the obtained results and the results reported by the SVM model, it can be affirmed that the support vector machine model could be suitable for determining the geographical origin of shiitake mushrooms.

### 3.3. Geographical Origin of Shiitake Mushrooms (Korean Origin-2 and Chinese Origin)

In this section, different models were developed to determine the origin of shiitake mushrooms in two categories: Korean origin-2 (218 replicates, made up of the two categories of Korean origin and Chinese inoculated) and Chinese origin-2 (60 replicates).

Once again, as in the previous sections, the three RF models (RF, RF_R_, and RF_Z_) showed the same accuracy and kappa values in all the phases. It seems to be verified, again, that, in this case, the normalization of the data does not affect the performance of the models. The RF model showed an accuracy value of 0.952 (kappa = 0.869) for the validation phase (Table 5). Notably, there was an improvement in the training phase compared to the validation phase, increasing to 0.986 for accuracy and 0.954 for kappa.

Attending to support vector machine models, all models achieved the same accuracy value (0.940), except for the SVM model and the SVM_Z_ model (both with 0.928). The difference between the best models lies in the kappa value, two of them with a value of 0.843. To select the best SVM model, the adjustments of the training and validation data have been used. In this case, the SVM_L_ shows a very slightly better accuracy (0.959) than the SVM_R_ model (0.955). Finally, the best artificial neural network model was the ANN_R_ model, which presented an accuracy of 0.978 (kappa = 0.930) and 0.940 (kappa = 0.843) for the training and validation phase, respectively.

In the testing phase, the RF and the ANN_R_ models showed the same accuracy value (0.893). The SVM model is the one that presents the worst results for this phase, with its accuracy value decreasing to 0.875 and the kappa value to 0.557.

Comparing the three selected best models, it can be said that the best model is the RF due to its highest accuracy value (0.952) in the validation phase (kappa value of 0.869), which places it in the upper part of the moderate level of the strength of agreement (according to the division established by Landis and Koch (1977) [57].

**Figure 4:**
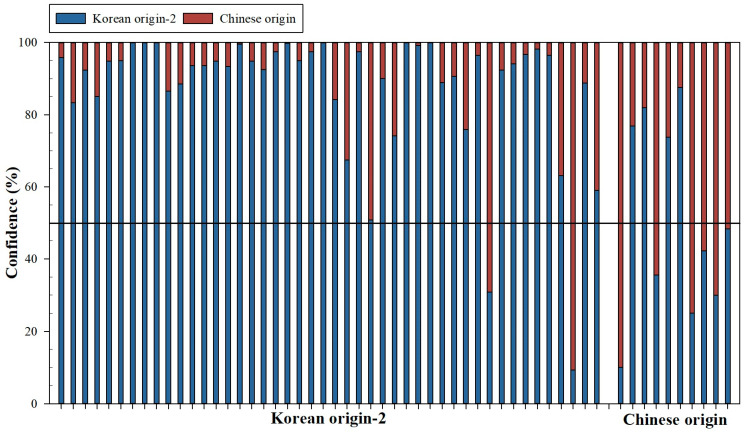
Confidence values (%) for test replicates obtained by the RF model.

Figure 4 shows the confidence level for the testing replicates using the RF model. The testing phase is made up of 56 replicates (46 Korean origin-2 and 10 Chinese origin).

Within the 46 Korean origin-2 replicates, 44 replicates have been correctly classified (30 of them with confidence values greater than 90.0%, 9 replicates with values between 75 and 90.0%, 4 replicates with values between 59.0 and 75.0%, and 1 replicate with a close confidence value of 50.8% (close to the wrong prediction)), and 2 replicates were wrongly classified, with confidence values of 69.2 and 90.7% for Chinese origin class. Related to the 10 Chinese origin replicates, it should be noted that 6 have been correctly classified with confidence values between 51.7 and 90.0%. The other 4 replicates of Chinese origin have been incorrectly classified with high confidence values for the Korean origin-2 class (between 87.5 and 73.8%).

The confidence matrix of the RF model for the test phase and all data used are shown in Table 4. In this case, the RF model showed an accuracy of 95.7 and 60.0% for the Korean origin-2 and Chinese origin categories, respectively. This model showed for all data an accuracy of 99.1 and 83.3% for the Korean origin-2 and Chinese origin categories, respectively. These results could be compared with the results obtained by Chung et al. (2021) [47] (Table 4), showing that the RF model obtained higher accuracy (95.7%) than the original model (82.0%) proposed by Chung et al. (2021) [47] for the original set.

Given the obtained results, and in agreeing with Chung et al. (2021) [47], it could be said that shiitake mushrooms grown in Korea using the Chinese inoculated sawdust blocks should be considered and labeled as Chinese origin due to the classification accuracies stated.

### 3.4. Final Considerations

As can be seen, the three different approximations made based on the grouping of the replicas (three categories or two categories, depending on where the data from the Chinese inoculated sawdust blocks are grouped) present good performance in all cases.

The best models selected within each cluster, based on the accuracy in the validation phase, showed that (i) two random forest models have turned out to be the best in two of the clusters (three categories and when the Chinese inoculated sawdust blocks replicates are referred to as Korean origin) and (ii) support vector machine is the best model to determine the origin in the clusters that groups Chinese inoculated sawdust blocks replicates as being of Chinese origin. These best models have presented, in the validation phase, accuracy values between 0.940 and 0.988, with high kappa values, all of them above 0.868. These good results were also extrapolated to the testing phase in which these three models presented accuracy values above 0.839 and kappa values above 0.603.

All these adjustments were obtained using a selection of hyperparameters based on the experience of the research group over the last ten years during the implementation of many models in the field of food technology and many others covering a variety from Physical Chemistry to Palynology. It is necessary to mention that the hyperparameters selection for the random forest method is based on the creation of sufficiently numerous trees in the forests (100) with deep trees (100)—carrying out pruning and pre-pruning to avoid overtraining of the trees—and whose decision criterion is not only the majority vote but also the greatest confidence in the prediction. The support vector machine hyperparameter selection was based on the guide prepared by Hsu et al. (2003) [53]. The steps between them were analyzed according to the author’s recommendations and in a logarithmic manner. Finally, hyperparameter selection for the artificial neural networks was based on carrying out the number of cycles necessary for them to pass the optimal training point. Likewise, decay was also used to modify the learning rate during training and thus favor the learning of the models.

Therefore, it could be said that the good performance shown by the selected models reported in this research has been the result of the parameter selection mentioned above. Despite this, it would be interesting to try to improve the results obtained here for the determination of the shiitake mushrooms’ origin considering the following options:

- Increase the number of replications for each of the assumptions analyzed (Korean, Chinese, and Chinese inoculated sawdust blocks);
- Analyze different distributions (%) in the training, validation and test groups;
- Study different normalization methods and normalization intervals for the data;
- Analyze different hyperparameters or increase their number, as well as analyze the increase and variation in the range and step of each of them;
- It would also be interesting to analyze the method of selecting the models using factors other than accuracy.

## 4. Conclusions

In this research, three supervised machine learning algorithms were carried out to model the geographical origin of shiitake cultivated in sawdust blocks. The highest accuracy and kappa values in the validation phase were the criteria used to choose the best models. The unnormalized RF and SVM models showed a greater generalization capacity and, at the same time, a greater performance in accuracy compared to the other developed models. In the testing phase, the ANN_R_ and SVM models developed to determine the shiitake origin into three categories presented the same values for accuracy and kappa (0.857 and 0.774, respectively); nevertheless, the accuracy and kappa values for the RF model were close, but were better in the validation phase. To classify the classification model into two categories (inoculated Chinese included as Chinese), the SVM presented the best generalization performance, with a high accuracy of 0.964 in the testing phase. Finally, when Chinese inoculated were included in the Korean replicates, the SVM presented lower results in precision and kappa values compared to RF and ANN_R_, which showed the same precision value (0.893). According to the results obtained, it can be said that the sawdust blocks inoculated in China and cultivated in Korea should be classified as having Chinese origin. The three machine learning algorithms (random forest, support vector machine, and artificial neural network) used in this research can be a promising tool to determine the origin of shiitake mushrooms. The developed models could be improved by increasing the number of replicas of each case analyzed by studying distributions, normalizations, and selection of different hyperparameters.

## Figures and Tables

**Table 1:** Adjustments for the best selected machine learning models developed to differentiate between each of the three categories (Korean origin, Chinese origin, and Chinese inoculated). The best model is presented in bold.

	Train		Validation		Training + Validation		Testing	
Model	Accuracy	Kappa	Accuracy	Kappa	Accuracy	Kappa	Accuracy	Kappa
**RF**	**0.971**	**0.954**	**0.940**	**0.908**	**0.959**	**0.936**	**0.839**	**0.742**
SVM	0.993	0.988	0.928	0.890	0.968	0.951	0.857	0.774
ANN_R_	0.971	0.954	0.940	0.908	0.959	0.936	0.857	0.774

**Table 2:** Confusion matrix obtained by the RF model on testing data and on all data. On the right are shown the values obtained by the model of Chung et al. (2021) [47]. In this table, K_O_ is Korean origin, C_O_ is Chinese origin, and C_i_ is Chinese inoculated. The asterisk indicates the results obtained by Chung et al. (2021) [47].

		Testing			All Data			All Data *		
		Predicted			Predicted			Predicted		
		K_O_	C_O_	C_i_	K_O_	C_O_	C_i_	K_O_	C_O_	C_i_
**True**	**K_O_**	23	0	0	123	1	1	114	8	3
	**C_O_**	2	6	2	6	49	5	4	26	30
	**C_i_**	3	2	18	3	2	88	2	13	78

**Table 3:** Adjustments for the best selected machine learning models developed to differentiate between Korean origin and Chinese origin-2 (Chinese origin and Chinese inoculated). The best model is presented in bold.

	Train		Validation		Training + Validation		Testing	
Model	Accuracy	Kappa	Accuracy	Kappa	Accuracy	Kappa	Accuracy	Kappa
RF	0.986	0.971	0.976	0.950	0.982	0.964	0.929	0.856
**SVM**	**0.993**	**0.986**	**0.988**	**0.975**	**0.991**	**0.982**	**0.964**	**0.927**
ANN_R_	0.986	0.971	0.976	0.951	0.982	0.964	0.893	0.787

**Table 4:** Confusion matrix obtained by the SVM and the RF model on testing data and on all data. On the right are shown the values obtained by the model of Chung et al. (2021) [47]. In this table, K_O_ is Korean origin, C_O_-2 is Chinese origin-2 (Chinese origin and Chinese inoculated), and K_O_-2 is Korean origin-2 (Korean origin and Chinese inoculated). The asterisk indicates the results obtained by Chung et al. (2021) [47].

		SVM Model						RF Model					
		Testing		All Data		All Data *		Testing		All Data		All Data *	
		Predicted		Predicted		Predicted		Predicted		Predicted		Predicted	
		K_O_	C_O_-2	K_O_	C_O_-2	K_O_	C_O_-2	K_O_-2	C_O_	K_O_-2	C_O_	K_O_-2	C_O_
**True**	**K_O_**	23	0	124	1	113	12	44	2	216	2	208	10
	**C_O_-2**	2	31	3	150	6	147	4	6	10	50	40	20

**Table 5:** Adjustments for the best selected machine learning models developed to differentiate between Korean origin-2 (Korean origin and Chinese inoculated) and Chinese origin. The best model is presented in bold.

	Train		Validation		Training + Validation		Testing	
Model	Accuracy	Kappa	Accuracy	Kappa	Accuracy	Kappa	Accuracy	Kappa
**RF**	**0.986**	**0.954**	**0.952**	**0.869**	**0.973**	**0.919**	**0.893**	**0.604**
SVM_L_	0.971	0.906	0.940	0.843	0.959	0.880	0.875	0.557
ANN_R_	0.978	0.930	0.940	0.843	0.964	0.894	0.893	0.635

## Data Availability

The data presented in this study are available on request from the corresponding authors. The data are not publicly available due to privacy restrictions.
